# Choroidal Haller's and Sattler's Layer Thickness Measurement Using 3-Dimensional 1060-nm Optical Coherence Tomography

**DOI:** 10.1371/journal.pone.0099690

**Published:** 2014-06-09

**Authors:** Marieh Esmaeelpour, Vedran Kajic, Behrooz Zabihian, Richu Othara, Siamak Ansari-Shahrezaei, Lukas Kellner, Ilse Krebs, Susanne Nemetz, Martin F. Kraus, Joachim Hornegger, James G. Fujimoto, Wolfgang Drexler, Susanne Binder

**Affiliations:** 1 Ludwig Boltzmann Institute of Retinology and Biomicroscopic Laser Surgery, Department of Ophthalmology, Rudolf Foundation Clinic, Vienna, Austria; 2 Center for Medical Physics and Biomedical Engineering, Medical University of Vienna, Vienna, Austria; 3 OptikNemetz, Vienna, Austria; 4 Pattern Recognition Lab and School of Advanced Optical Technologies (SAOT), Friedrich-Alexander University Erlangen-Nuremberg, Erlangen, Bavaria, Germany; 5 Department of Electrical Engineering and Computer Science, Massachusetts Institute of Technology, Cambridge, Massachusetts, United States of America; Medical University of South Carolina, United States of America

## Abstract

**Objectives:**

To examine the feasibility of automatically segmented choroidal vessels in three-dimensional (3D) 1060-nmOCT by testing repeatability in healthy and AMD eyes and by mapping Haller's and Sattler's layer thickness in healthy eyes

**Methods:**

Fifty-five eyes (from 45 healthy subjects and 10 with non-neovascular age-related macular degeneration (AMD) subjects) were imaged by 3D-1060-nmOCT over a 36°x36° field of view. Haller's and Sattler's layer were automatically segmented, mapped and averaged across the Early Treatment Diabetic Retinopathy Study grid. For ten AMD eyes and ten healthy eyes, imaging was repeated within the same session and on another day. Outcomes were the repeatability agreement of Haller's and Sattler's layer thicknesses in healthy and AMD eyes, the validation with ICGA and the statistical analysis of the effect of age and axial eye length (AL) on both healthy choroidalsublayers.

**Results:**

The coefficients of repeatability for Sattler's and Haller's layers were 35% and 21% in healthy eyes and 44% and 31% in AMD eyes, respectively. The mean±SD healthy central submacular field thickness for Sattler's and Haller's was 87±56 µm and 141±50 µm, respectively, with a significant relationship for AL (P<.001).

**Conclusions:**

Automated Sattler's and Haller's thickness segmentation generates rapid 3D measurements with a repeatability correspondingto reported manual segmentation. Sublayers in healthy eyes thinnedsignificantly with increasing AL. In the presence of the thinned Sattler's layer in AMD, careful measurement interpretation is needed. Automatic choroidal vascular layer mapping may help to explain if pathological choroidal thinning affects medium and large choroidal vasculature in addition to choriocapillaris loss.

## Introduction

The choroid is a vascular structure that provides the outer retina with oxygen and nutrition [1], [2]. Most of the choroidal space is taken by vessels differentiated in three vascular layers that are markedly defined by their location and lumen size [3]–[5]. In the healthy eye, the choriocapillaris are a continuous network bordering Bruch's membrane. They branch from the medium- and small-sized vessels in the Sattler's layer. Haller's layer is the outermost layer and consists of posterior ciliary arteries and large lumen veins. All three sublayers are thickest at the posterior pole with many branches and larger vessel calibers in the Sattler's and Haller's layer. Total choroidal thickness is reduced with advanced age[6]–[8] and in prominent outer retinal pathologies such as in age-related macular degeneration (AMD)[9], [10]. Pathological choroidal alterations include the reduced choroidal vessel density associated with sub-retinal pigment epithelium (RPE) deposits[11] and the fibrosis observed in AMD and subretinal deposits (reticular pseudodrusen)[12]. The role of the subretinal deposits in relation to the choroid is still under investigation. Choroidal thinning indicating atrophy has been observed after anti-vascular endothelial growth factor treatments of neovascular AMD[13]. The choriocapillaris layer accounts only for a small proportion of the total choroidal thickness in the healthy eye.[14]Choriocapillaris density decrease in relation to aging, and AMD[11], [15], [16] explainsthe choroidal pathogenesis in AMD only partially. Choroidal involvement in posterior ocular disease may be further explained by thickness quantification of the medium and large choroidal vessels of the Sattler's and Haller's layers.

Optical coherence tomography (OCT) used with a long wavelength light source of 1060 nm can visualize the choroid and the vasculature of Sattler's and Haller's layers in healthy eyes and AMD eyes [17], [18]. An unprecedented automated blood vessel segmentation algorithm has been developed that allows segmentation and 3-dimensional (3D) visualization of the choroidal vessels in OCT images[19]. The current study tested the repeatability of vessel segmentation in healthy and AMD eyes. The vessel segmentation was validated by comparing enface OCT vessel segmentations to indocyanine green angiography (ICGA) images in eyes with AMD. The feasibility of Haller's from Sattler's layer thickness maps was examined in healthy eyes. The clinical measurement utility was improved by introducing averaging across the Early Treatment Diabetic Retinopathy Study (ETDRS) grid.

## Methods

A single-institution, prospective, cross-sectional study of Sattler's and Haller's layer thickness was conducted. Ethical approval was obtained prospectively from the local Institutional Review Board, ”The Ethical Commission of Vienna.” This study was in adherence to the tenets of the Declaration of Helsinki. The subjects agreed to participate after explanation of the nature and possible consequences of the study. Written informed consent was obtained for experimentation with human subjects prior to participation.

### Subjects

Overall, fifty-five subjects consisting of forty-five healthy subjects and ten subjects with non-neovascular AMD were recruited to test the repeatability and validity of the segmentation algorithm and the distribution of the Haller's and Sattler's thickness measurements.

For the statistical analysis of vessel distribution, Haller's and Sattler's layer thicknesses were measured in forty-five healthy subjects (27 females, age range 23 to 84 years, mean±SD 44±16 years) with no history of retinal surgery or pathology and no cataract surgery. Subjects with myopia >6 diopters (or axial eye length>26.5 mm) and smokers were excluded. All subjects had best corrected visual acuities (BCVA) of 20/20 or better (0 LogMAR or less on the ETDRS chart),whereas two subjects with cataracts and a visual acuity of 20/32 Snellen (0.2 LogMAR) were included based on the clinical appearance of healthy retinas. Only one eye per healthy subject was included. All subjects had no history of high blood pressure nor were they taking any medications for hypertension (blood pressure was taken within the trial day to confirm health status). Five axial eye length (AL) measurements were averaged from each eye using optical biometry (IOL Master Zeiss, Jena, Germany). AL was investigated as an independent factor that may relate to vessel layer thickness. For the healthy cohort, AL had a mean±SD of 23.76±1.1 mm with a range of 22.12 to 26.15 mm.

To evaluate the repeatability of the vessel segmentation, a total of twenty eyes from twenty subjects were reimaged at the same session and on another trial day within two months of the first imaging, resulting in 60 measurements. The repeatability cohort consisted of ten randomly selected healthy subjects (age range 23–45 years, 6 females, two with long ALs and moderate myopia and two with short ALs and moderate hyperopia) and ten patients with non-neovascular AMD in the study eye and neo-vascular AMD in the fellow eye. The patients had a mean visual acuity of 20/20 on both visits (0 LogMar with a range between 0.2 and −0.16). Fundus examination was performed after pupil dilation by an experienced ophthalmologist with slit lamp biomicroscopy and a Volk lens. All included AMD subjects had no cataracts, and clinical examination of the fundus was possible. According to the current classification guidelines[20], the AMD cohort consisted of low to severe AMD stages (two eyes with pigment changes, seven eyes with drusen, and one eye with geographic atrophy, age range 67–80 years, 5 females). Three of the seven eyes with drusen had areas of subretinal deposits. This range of AMDs allowed the investigation of varying signal to noise ratios. To validate vessel segmentation and to assess its resolution,ICGA images were used. Five eyes from five subjects with no cataracts were subjected to ICGA, which was needed for their clinical routine examination.

### OCT imaging

**Figure 1:**
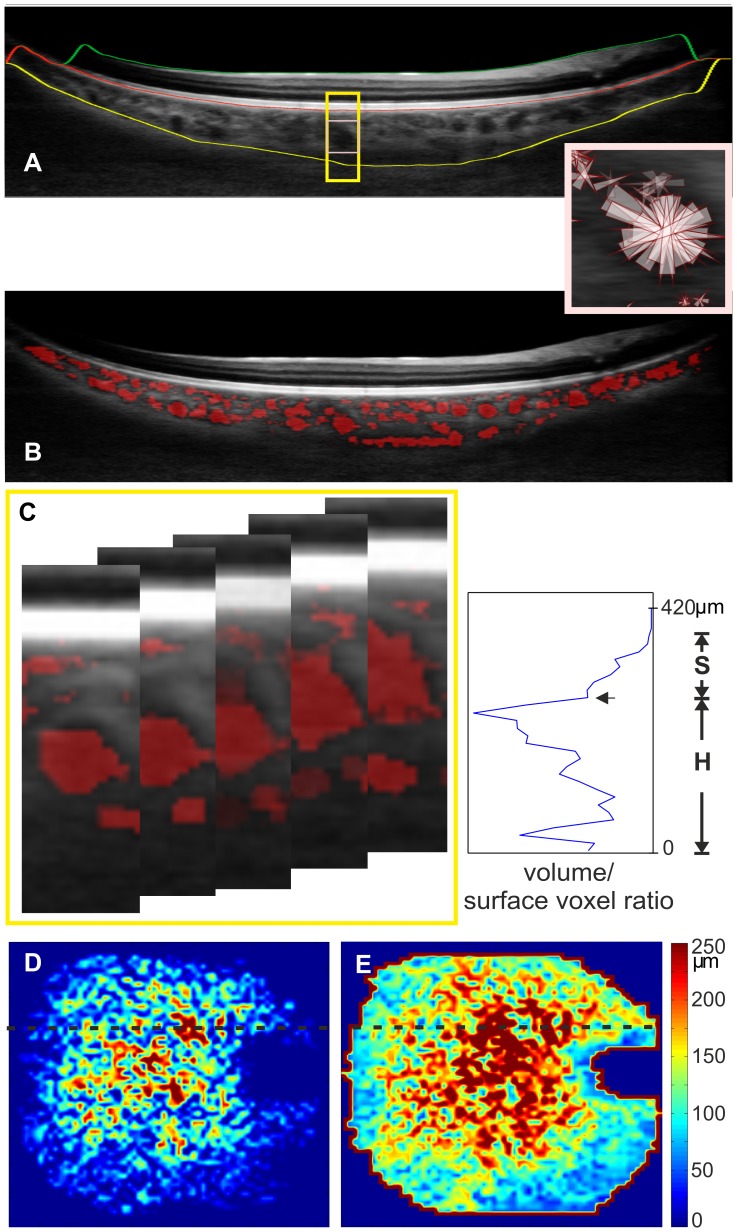
Automatic segmentation of Haller's and Sattler's layer for thickness mapping. OCT B-scan (A) visualizes the segmentation lines for the retina between the ILM (green line) and the RBC complex and the identified vessel voxels are visualized in red (B). The choroid is located between the RBC complex and the sclera (yellow line). Probability cones (small rectangle enlarged) were drawn to demonstrate expected vessel core position. The cone projections are in 3D, butthe concept is presented in two dimensions. The ratio of volume voxels to surface voxels that were generated was plotted for a 1×1 degree volume. This volume consists of 14 B-scans and is represented by the 5 B-scans in the yellow rectangle (C). The plot is used for determining the border between the two sublayers by detecting the largest ratio as Haller's layer vessels (H),and the local minimum is the border (arrow) to Sattler's layer (S). Plots were used to generate thickness maps for Sattler's (D) and Haller's layers (E) across the scan area.

High speed, 56.000 A-scans/s, spectral domain OCT (SD-OCT) imaging at 1060 nm was performed with less than 2.5 mW at the cornea, which is well below the maximum power limit for a 10-s exposure[21], [22]. 3D OCT volumes were acquired at 1060 nm with a 15–20- µm transverse resolution, approximately 7- µm axial resolution and 512 voxels per depth-scan (A-scan) (Figure 1 A). Raster scans consisting of 512 A-scans and 512 B-scans were acquired across a 36×36° field in 4.68 seconds. The data processing procedure for choroid vessel segmentation has been described elsewhere[19]. Briefly, two orthogonal volumes were used to estimate retinal motion by associating a displacement field with each volume and calculating the displacements, while optimizing similarity and penalizing motion on an A-scan basis. Motion corrected 3D OCT datasets were then merged to create a 3D dataset with improved signal and reduced speckle[23]. The “Neat Video” plugin in VirtualDub (Free Software Foundation, Inc., Cambridge, MA, USA) for denoising was applied to reduce speckle.

### Total choroid and choroidal vessel segmentation

Data segmentation was performed in the following order: automatic choroid segmentation [24], [25], automatic vessel segmentation within the choroid, and subsequent automatic layer segmentation. Automatic choroidal segmentation was used to define the region for choroid vessel segmentation. Accordingly, axial choroidal thickness was defined as the distance between the retinal pigment epithelium/Bruch's membrane/choriocapillaris (RBC) complex and the choroidal-scleral interface (Figure 1 A). The pixel distance was converted into optical distance using the depth sampling calibration for the 1060-nm OCT system and further to the anatomical distance. Within this region, novel automated vessel segmentation was performed[19]. This method determines vessel cores by projecting probability cones to avoid relying on the poorly defined vessel wall (Figure 1 A). The probability cones are projected in three dimensions, andusing 3D filtering, the estimated vessel core was dilated to approach the full vessel diameter. For the comparison of choroid vessel segmentations to ICGA images, vessel segmentations were viewed en face at different choroid depths.

### Sattler's and Haller's layer segmentation

Sattler's and Haller's layers are defined as medium-size and large-size vessel layers, respectively. For determining the border between both layers, Haller's layer was defined as the layer containing the largest vessel. Vessel sizes were differentiated by using the ratio of the voxels within the vessel volume to the voxels at its surface. In general, smaller vessels have a smaller ratio than larger vessels. The ratio was plotted versus the choroidal depth and contained the ratio of a 1×1 degree volume (Figure 1 B). On the plot, Haller's layer was started from the scleral boundary, including the highest voxel ratio until the first small ratio on the plot. Therefore, this first valley after the plot's maximum marked the border between Haller's and Sattler's layer. The inner border of Sattler's layer was determined by detecting no vessels or noise, such as vessels consisting of a single voxel. Plot representations at the subfoveal choroid were qualitatively compared to actual vessel depiction by OCT and corresponded to vessel distribution in this location. Plots across the 36×36 degree field of view were used to generate Sattler's and Haller's layer thickness maps (Figures 1 D and 1 E, respectively) with Matlab software (The MathWorks, Inc., Natick, USA).

### Outcome measures

The main outcome was the agreement between the repeatedly measured Sattler's and Haller's layer thicknesses in healthy and non-neovascular AMD. The secondary outcomes were the agreement of vessel segmentation with ICGA and the mean ± standard deviation of the choroidalsublayers expressed for the nine subfields of the ETDRS grid in a healthy eye cohort.

### Statistical evaluation

**Figure 2:**
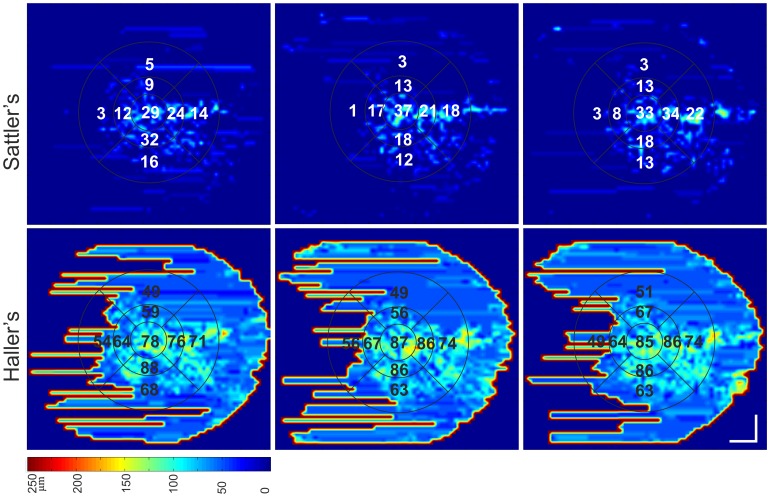
Repeatability of Sattler's and Haller's layer thickness mapping and ETDRS measurements in non-neovascular AMD. Sattler's layer and Haller's layer are color mapped. Imaging was performed twice at the same session (left and middle column) and on a different day (right column). The vessel segmentation and indocyanine green angiography images of this eye are depicted in Figure 3.

The central submacular field (CSM) and the total macular field, with a diameter of 1.5 and 6 mm, respectively, were statistically analyzed for the repeatability testing. The difference was calculated between the same session and different day imaging and their confidence intervals (CI). To assess a possible relationship of the variability with the magnitude of the mean Bland-Altman plots for each group, each sublayer was plotted at the CSM and total macular field.[26] The 95% limits of agreement for small groups were found by multiplying the ±standard deviations by 2. To assess reliability, intraclass correlations (ICC) with measures of absolute agreement and their CIs were calculated. The coefficient of repeatability was calculated for the absolute and relative differences of repeated imaging.[27] Maps were qualitatively compared for repeatability between the three measurements (Figure 2).

The statistics software IBM SPSS Statistics for Windows, Version 20.0 (IBM Corp., Armonk, NY) was used for conducting normality testing, repeatability testing, ANOVA testing and a multiregression analysis of the possible contribution of independent variables (age, AL, refraction and gender) to choroidalsublayer thickness. Central measurements for statistical analysis were performed beneath the foveola.

## Results

### Repeatability of vessel segmentation

The average sublayer thickness measurements and their differences for the two groups obtained at each session are presented in Table 1. Intraclass correlations and coefficients of repeatability are presented in Table 2 and should be interpreted with respect to the mean measurements in Table 1.

**Table 1:** Average repeated choroidalsublayer thickness measurements for the central submacular field (CSM) and the total macular field ( µm).

Layer	R1 (range)	R2 (range)	R3 (range)	*P* value	Δ R1-R2 (CI, LoA)	Δ R1-R3(CI, LoA)
Healthy						
Sattler's (CSM)	116±66(10–217)	114±69(10–218)	123±68(6–225)	.17	2 (−8–13, 59)	−7(−18–5, 64)
Sattler's (total)	98±52(8–196)	94±508–176)	102±58(14–220)	.6	2 (−2–8, 32)	−4 (−12–2, 39)
Haller's (CSM)	226±80 (89–348)	228±86(87–361)	216±85 (86–382)	.54	1(−6–7, 31)	−4(−24–16, 66)
Haller's (total)	180±46(79–241)	184±51(78–253)	173±38 (63–228)	.2	−4 (−12–4, 45)	9(−13–30, 49)
Eyes with non-neovascular AMD						
Sattler's (CSM)	20±4(4–99)	20±4(2–88)	22±4 (5–133)	.61	−1 (−0.8–1.4, 17)	−1 (−1.5–1, 6)
Sattler's (total)	10±3(2–46)	9±3(2–40)	12±3(4–50)	.62	−1 (−0.9–1.4, 6)	0 (0−1, 5)
Haller's (CSM)	139±45 (80–234)	144±33 (82–233)	149±38 (82–219)	.37	−3(−14–9, 65)	−6 (−14–3, 50)
Haller's (total)	95±27(61–147)	93±27(57–151)	90±21(62–133)	.37	2(−3–8, 31)	3 (−5–10, 43)

R1 and R2 represent imaging at the same session. R3 was performed on a different day. P values are stated for the difference between measurements by repeated measures ANOVA. Δ represents the difference. CI is the 95% confidence interval. LoA represents 95% limits of agreement. Data are expressed as the mean ± standard deviation.

**Table 2:** Intraclass correlations (ICC) and coefficients of repeatability (CR).

Layer	ICC R1-R2 (CI)	ICC R1-R3 (CI)	CR R1-R2	CR R1-R3
Healthy				
Sattler's (CSM)	0.99 (0.96–1)	0.95 (0.94–1)	40 (35%)	32 (27%)
Sattler's (total)	0.96 (0.84–0.99)	0.99 (0.96–1)	22 (23%)	26 (26%)
Haller's (CSM)	0.99 (0.98–1)	0.97 (0.88–0.99)	22 (10%)	46 (21%)
Haller's (total)	0.99 (0.96–1)	0.98 (0.89–0.99)	32 (18%)	33 (19%)
Non-neovascular AMD				
Sattler's (CSM)	0.98 (0.93–0.99)	0.86 (0.44–0.97)	4 (20%)	4 (20%)
Sattler's (total)	0.95 (0.82–0.99)	0.96 (0.86–0.99)	4 (44%)	4 (34%)
Haller's (CSM)	0.9 (0.59-0-98)	0.92 (0.69–0.98)	44 (31%)	34 (23%)
Haller's (total)	0.97 (0.9–0.99)	0.95 (0.80–0.99)	21 (22%)	19 (21%)

R1 and R2 represent imaging at the same session. R3 was performed on a different day. CI is the 95% confidence interval. CR represents the coefficient of repeatability, expressed in parenthesis as the percentage of the mean difference.

The measurement of the total macular field repeatability was similar to measuring repeatability in the CSM field alone. The coefficients of repeatability were 35% and 21% in healthy eyes and 44% and 31% in AMD eyes for Sattler's and Haller's layers, respectively. The Sattler's layer was very thin in five of the ten AMD eyes. These eyes had a Sattler's layer thickness of <20 µm for the CSM subfield and <10 µm for the total macular field. In this small range of thickness measurements, the absolute differences yielded a high coefficient of repeatability. Sattler's and Haller's maps displayed similar thickness values and thickness distributionswhen eachrepeated imaging resultwas compared (Figure 2).

No significant difference was detected between repeated imaging (*P* values in Table 1), and no significant association between the thickness difference and the thickness mean in each group and in each choroidalsublayer was observed on the Bland-Altman plots. The ICCs indicated a strongagreement between the repeated measurements.

### Validation of vessel segmentation with indocyanine green angiography

**Figure 3:**
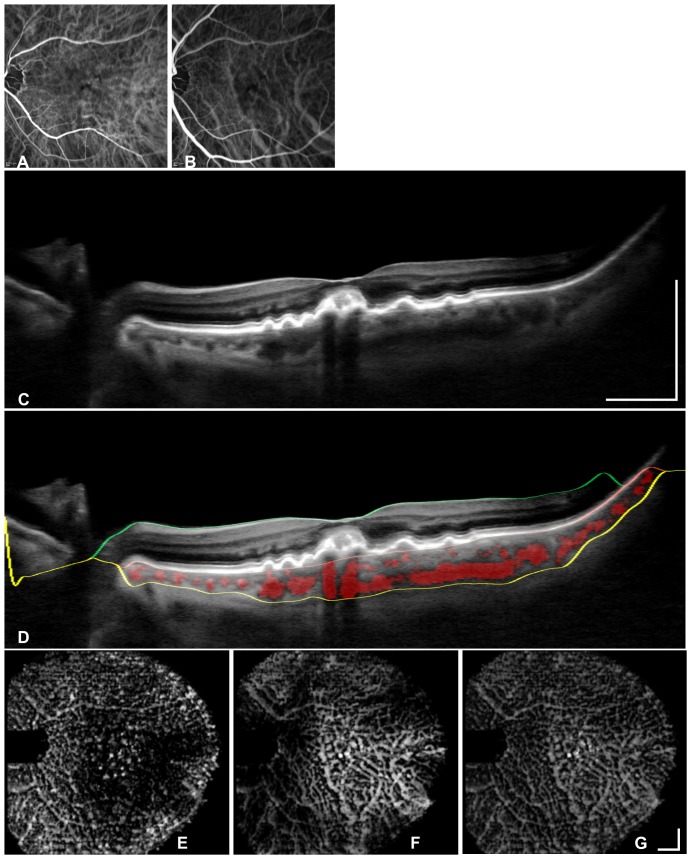
Segmentation validation in a non-neovascular AMD eye via a comparison of the segmented vasculature with indocyanine green angiography (ICGA). Early phase (A) and middle phase (B) angiography images show the ICGAresolution for choroidal vascular imaging. OCT B-scan centered on the fovea (C), and segmented areasare marked in red (D) to visualize vessel segmentation. The OCT B-scan (D) shows the segmentation lines for the retina between the ILM (green line) and the RBC complex. The choroid is located between the RBC complex and the sclera (yellow line). En face views are locatedat the inner choroid (E), the outer choroid (F) and the entire choroid (G) to demonstrate captured vessels and depth information. The bars represent 1 mm.

ICGA images of the non-neovascular AMD eyes (Figure 3) display a segmental vessel pattern with many large watershed zones in both phases. The enface OCT images (Figures 3 bottom row) visualize the same vessels with a comparably similar resolution to the ICGA images.

### Healthy choroidal vessel segmentation and layer thicknesses

The mean layer thicknesses for each ETDRS macular subfields of Haller's and Sattler's layer for the healthy eye cohort are stated in Table 3. Multiregression analysis indicated a significant statistical relationship for each Haller's and Sattler's layer central subfield with age and AL (Sattler's layer: *R* ^2^ = 0.24, *P* = .003, AL: Beta = −48 *P*<.001, age Beta = −28 *P* = .05 and Haller's layer: *R* ^2^  = 0.39, *P*<.001, AL: Beta = −62 *P*<.001, age Beta = −30 *P* = .02). The two independent factors AL and refraction displayed a linear association with each other (collinearitydiagnostics testing of multiregression analysis). Refraction had a weaker effect than AL on Sattler's and Haller's layers (Beta = −38 and −44, respectively) and was excluded from the analysis. Therefore, despite the high variability, the relationship between AL and Sattler's and Haller's layer in the healthy cohort of 45 eyes was statistically significant, and the relationship-related variation exceededthe imprecision inherent to the methodology (Table 3).

**Table 3:** Automated calculated mean for healthy Sattler's and Haller's layer thickness across the Early Treatment Diabetic Retinopathy Study grid ( µm).

Subfield	Sattler's layer	Haller's layer
central submacular	87±56 (3–264)	141±50 (57–237)
superior inner	77±51 (13–268)	144±44 (65–251)
temporal inner	71±52 (3–233)	131±53 (48–255)
inferior inner	77±55 (4–246)	133±47 (48–225)
nasal inner	81±60 (1–240)	133±38 (58–211)
superior outer	74±45 (10–243)	121±33 (70–195)
temporal outer	50±45 (4–213)	103±40 (50–181)
inferior outer	65±49 (5–215)	117±37 (64–180)
nasal outer	64±50 (3–203)	110±35 (66–166)
Grouped by axial eye length for statistical analysis for the CSM subfield^a^		
Hyperope n = 15	114±64 (3–264)	168±43 (57–236)
Emmetrope n = 18	79±45 (19–165)	133±46 (70–230)
Myope n = 12	48±35 (3–99)	104±49 (50–236)
*P* value^b^	.006 (.004)	.003 (.002)
Grouped by age (years) for the CSM subfield		
< 45 n = 27	91±51	144±50
>45 n = 18	70±59	125±52

Data are expressed in µm and as the mean ± standard deviation (range).

a. Eyes were grouped based on the normal AL variation with refraction and age by myopia: AL≥24.5 mm, emmetropic: 24.5>AL≥23.4 mm or hyperopic:AL<23.4 mm)

b. univariate ANOVA (post-hoc test Tukey for hyperopic and myopic eyes)

## Discussion

Different approaches to *in vivo*choroidal vessel analysis have been suggested in the literature. Choroidal vessel visualization using angiography has shown the segmental vessel structure and their respective watershed zones[28], [29]. Watershed zones are highly affected by ageing and ischemia and may offer a more sensitive measure of pathological changes than choroidal thickness change alone [30]. Choroidal OCT imaging offers noninvasive vessel visualization. OCT pixel intensitymeasurementshave been suggested for the analysis of choroidal vessels in AMD eyes [31]. However, this method has been used without determining the border between Sattler's and Haller's layers and may be unfeasible when the OCT images are affected by retinal and RPE lesions. Motaghiannezam et al. demonstrated an algorithm for automated OCT vessel segmentation in three healthy eyes [32], whereas Zhang et al. investigated a cohort of twenty-four healthy eyes, but they could not successfully reproduce repeat automated segmentation in all eyes[33]. In this study, the segmentation of vessels and Sattler's and Haller's layer was successfully applied in all healthy and pathological eyes. Furthermore, the automatic vessel segmentation was validated against ICGA and captured most choroidal vessels despite non-neovascular AMD lesions.

This study found a significant relationship with AL, and the relationship-related variationexceeded the segmentation variability. Healthy sublayer thinning with age was significant,but the level of thinning was below the measurement variability. For every 10 years of ageing,14 to 15 µm of choroidal thinning has been reported [6], [8], and choroidal thinning is further increased in the presence of AMD phenotypes, such as in eyes with subretinal deposits[12] or with geographic atrophy[34]. Future research is needed to explore if pathological choroidal thinning is related to Sattler's and Haller's layers with a larger thickness difference than the coefficient of repeatability of this study.

Automatic segmentation precision is defined by measurement repeatability and is dependent on the visualization quality of the interfaces. For choroidal thickness measurements, the quality is determined by the signal to noise ratio of the OCT image. The signal is reduced with increasing depth or by image degradation below a retinal lesion,whereas depth has a stronger effect on the measurement variability [35]. Healthy eyes have commonly thick choroids, and the current study demonstrated that automatic vessel segmentation was not deteriorated in these eyes. A further source for imprecision is the noise caused by the inconsistency of positioning. In the AMD cohort, the signal may have been further reduced by unstable fixation. Some AMD eyes had a very thin Sattler's layer; therefore,small deviations resulted in high coefficients of repeatability. As such, the coefficients of repeatability need to be viewed in relation to the absolute measurements, and a large AMD cohort is needed to evaluate if a thin Sattler's layer correlates to the clinical appearance.

The choroid displays a high variability, which is reflected in large standard deviations when compared to the retina and its sublayers[25], [36]. The choroidal vasculature is even more challenging. When a vessel's course is parallel to the OCT scan, the cross-section of the vessel does not appear round. This complexity reduces manual vessel segmentation precision, which is performed in two-dimensional scans. To improve manual segmentation repeatability, training is needed ideally [37] whereas there is no training or expertise needed for automatic segmentation after the initial validation. Manual segmentation in diabetic eyes has demonstrated 26% and 32% repeatability for Sattler's layer and 30% and 32% for Haller's layer for the central submacular field and total macular field, respectively [37]. The automatic segmentation in this paper displayed a comparably good variability for healthy and diseased eyes with a range of different choroidal thicknesses and noise distributions.

In conclusion, this study utilized a rapid and clinically feasible method to obtain the spatial distribution of choroidalsublayers with 3D 1060-nm OCT mapping and with the widely used ETDRS grid subfields. Sublayer variation with AL was greater thanthe imprecision of the methodology, and mapping allowed for the observation of the sublayer's thicknessover the scanning field. To the best of our knowledge, the present choroidalsublayer segmentation offers the most repeatable and reliable automated qualitative and quantitative analysis method for clinical use and the investigation of pathological choroidal thinning. For future studies of Sattler's and Haller's layers and for the clinical application of vessel segmentation in AMD eyes, the variability caused by noise and the morphological complexity of choroidal vessels need to be considered.

## References

[1] Bill A, Sperber G, Ujiie K (1983) Physiology of the choroidal vascular bed. Int Ophthalmol 6: 101–107. 6403480 10.1007/BF00127638

[2] Linsenmeier RA, Padnick-Silver L (2000) Metabolic dependence of photoreceptors on the choroid in the normal and detached retina. Invest Ophthalmol Vis Sci 41: 3117–3123. 10967072

[3] Hayreh SS (1974) The choriocapillaris. Albrecht von Graefes Arch Klin Ophthalmol 192: 165–179. 10.1007/BF00416864 4219556

[4] Zhang HR (1994) Scanning electron-microscopic study of corrosion casts on retinal and choroidal angioarchitecture in man and animals. Progress in Retinal and Eye Research 13: 243–270.

[5] Nickla DL, Wallman J (2010) The multifunctional choroid. Prog Retin Eye Res 29: 144–168. 20044062 10.1016/j.preteyeres.2009.12.002 PMC2913695

[6] Margolis R, Spaide RF (2009) A pilot study of enhanced depth imaging optical coherence tomography of the choroid in normal eyes. Am J Ophthalmol147: 811–815. 10.1016/j.ajo.2008.12.008 19232559

[7] Spaide RF (2009) Age-related choroidal atrophy. Am J Ophthalmol147: 801–810. 10.1016/j.ajo.2008.12.010 19232561

[8] Ikuno Y, Kawaguchi K, Yasuno Y, Nouchi T (2009) Choroidal Thickness in Healthy Japanese Subjects. Invest Ophthalmol Vis Sci51: 2173–2176. 10.1167/iovs.09-4383 19892874

[9] Manjunath V, Goren J, Fujimoto JG, Duker JS (2011) Analysis of choroidal thickness in age-related macular degeneration using spectral-domain optical coherence tomography. Am J Ophthalmol 152: 663–668. 21708378 10.1016/j.ajo.2011.03.008 PMC3375176

[10] Koizumi H, Yamagishi T, Yamazaki T, Kawasaki R, Kinoshita S (2011) Subfoveal choroidal thickness in typical age-related macular degeneration and polypoidal choroidal vasculopathy. Graefes Arch Clin Exp Ophthalmol 249: 1123–1128. 21274555 10.1007/s00417-011-1620-1

[11] Mullins RF, Johnson MN, Faidley EA, Skeie JM, Huang J (2011) Choriocapillaris vascular dropout related to density of drusen in human eyes with early age-related macular degeneration. Invest Ophthalmol Vis Sci 52: 1606–1612. 21398287 10.1167/iovs.10-6476 PMC3101687

[12] Querques G, Querques L, Forte R, Massamba N, Coscas F, et al (2012) Choroidal changes associated with reticular pseudodrusen. Invest Ophthalmol Vis Sci 53: 1258–1263. 22222508 10.1167/iovs.11-8907

[13] Young M, Chui L, Fallah N, Or C, Merkur AB, et al.. (2014) Exacerbation of choroidal and retinal pigment epithelial atrophy after anti-vascular endothelial growth factor treatment in neovascular age-related macular degeneration. Retina. doi:10.1097/IAE.0000000000000081. 10.1097/IAE.0000000000000081 24451923

[14] Hogan MJ, Alvarado JA, Weddell JE (1971) Histology of the human eye: an atlas and textbook. Hogan MJ, Alvarado JA, Weddell JE, editors. Saunders. pp 322–371.

[15] Ramrattan RS, van der Schaft TL, Mooy CM, de Bruijn WC, Mulder PG, et al (1994) Morphometric analysis of Bruch's membrane, the choriocapillaris, and the choroid in aging. IOVS 35: 2857–2864. 8188481

[16] McLeod DS, Taomoto M, Otsuji T, Green WR, Sunness JS, et al (2002) Quantifying changes in RPE and choroidal vasculature in eyes with age-related macular degeneration. Invest Ophthalmol Vis Sci 43: 1986–1993. 12037009

[17] Unterhuber A, Považay B, Hermann B, Sattmann H, Chavez-Pirson A, et al (2005) In vivo retinal optical coherence tomography at 1040 nm-enhanced penetration into the choroid. Opt Express 13: 3252–3258. 19495226 10.1364/opex.13.003252

[18] De Bruin DM, Burnes DL, Loewenstein J, Chen Y, Chang S, et al (2008) In vivo three-dimensional imaging of neovascular age-related macular degeneration using optical frequency domain imaging at 1050 nm. Invest Ophthalmol Vis Sci 49: 4545–4552. 18390638 10.1167/iovs.07-1553

[19] Kajic V, Esmaeelpour M, Glittenberg C, Kraus MF, Honegger J, et al (2013) Automated three-dimensional choroidal vessel segmentation of 3D 1060 nm OCT retinal data. Biomed Opt Express 4: 134–150. 23304653 10.1364/BOE.4.000134 PMC3539191

[20] Ferris FL 3rd, Wilkinson CP, Bird A, Chakravarthy U, Chew E, et al (2013) Clinical classification of age-related macular degeneration. Ophthalmology 120: 844–851. 23332590 10.1016/j.ophtha.2012.10.036 PMC11551519

[21] ANSI (2000) Safe Use of Lasers & Safe Use of Optical Fiber Communications. American National Standard Institute - Z136 Committee. 168 p.

[22] ICNIRP (2000) Revision of the Guidelines on Limits of Exposure to Laser radiation of wavelengths between 400nm and 1.4 µm. International Commission on Non-Ionizing Radiation Protection. 431–440 p. 11007467

[23] Kraus MF, Potsaid B, Mayer MA, Bock R, Baumann B, et al (2012) Motion correction in optical coherence tomography volumes on a per A-scan basis using orthogonal scan patterns. Biomed Opt Express 3: 1182–1199. 22741067 10.1364/BOE.3.001182 PMC3370961

[24] Kajic V, Esmaeelpour M, Považay B, Marshall D, Rosin PL, et al (2011) Automated choroidal segmentation of 1060 nm OCT in healthy and pathologic eyes using a statistical model. Biomed Opt Express 3: 86–103. 22254171 10.1364/BOE.3.000086 PMC3255345

[25] Esmaeelpour M, Povazay B, Hermann B, Hofer B, Kajic V, et al (2010) Three-dimensional 1060nm OCT: Choroidal thickness maps in normals and improved posterior segment visualization in cataract patients. Invest Ophthalmol Vis Sci 51: 5260–6. 20445110 10.1167/iovs.10-5196

[26] Bland JM, Altman DG (1986) Statistical methods for assessing agreement between two methods of clinical measurement. Lancet 1: 307–310. 2868172

[27] Bland JM, Altman DG (1996) Statistical methods for assessing agreement between two methods of clinical measurement. Lancet 1: 307–310. 2868172

[28] Hayreh SS (1990) In vivo choroidal circulation and its watershed zones. Eye (Lond) 4 (Pt 2): 273–289. 10.1038/eye.1990.39 2199236

[29] Hayreh SS (2004) Posterior Ciliary Artery Circulation in Health and Disease The Weisenfeld Lecture. Invest Ophthalmol Vis Sci 45: 749–757. 14985286 10.1167/iovs.03-0469

[30] Ito YN, Mori K, Young-Duvall J, Yoneya S (2001) Aging changes of the choroidal dye filling pattern in indocyanine green angiography of normal subjects. Retina 21: 237–242. 11421013 10.1097/00006982-200106000-00007

[31] Sohrab M, Wu K, Fawzi AA (2012) A Pilot Study of Morphometric Analysis of Choroidal Vasculature In Vivo, Using En Face Optical Coherence Tomography. PLoS ONE 7: e48631 doi:10.1371/journal.pone.0048631 23189132 10.1371/journal.pone.0048631 PMC3506620

[32] Motaghiannezam R, Schwartz DM, Fraser SE (2012) In Vivo Human Choroidal Vascular Pattern Visualization Using High-Speed Swept-Source Optical Coherence Tomography at 1060 nm. Invest Ophthalmol Vis Sci 53: 2337–2348. 22410568 10.1167/iovs.11-7823

[33] Zhang L, Lee K, Niemeijer M, Mullins RF, Sonka M, et al (2012) Automated segmentation of the choroid from clinical SD-OCT. Invest Ophthalmol Vis Sci 53: 7510–7519. 23060139 10.1167/iovs.12-10311 PMC3490539

[34] Lee JY, Lee DH, Lee JY, Yoon YH (2013) Correlation between subfoveal choroidal thickness and the severity or progression of nonexudative age-related macular degeneration. Invest Ophthalmol Vis Sci 54: 7812–7818 doi:–10.1167/iovs.13–12284 24204054 10.1167/iovs.13-12284

[35] Kim JH, Kang SW, Kim JR, Kim SJ (2013) Variability of subfoveal choroidal thickness measurements in patients with age-related macular degeneration and central serous chorioretinopathy. Eye (Lond) 27: 809–815. 23598679 10.1038/eye.2013.78 PMC3709398

[36] Ikuno Y, Maruko I, Yasuno Y, Miura M, Sekiryu T, et al (2011) Reproducibility of Retinal and Choroidal Thickness Measurements in Enhanced Depth Imaging and High-Penetration Optical Coherence Tomography. Invest Ophthalmol Vis Sci 52: 5536–5540. 21508114 10.1167/iovs.10-6811

[37] Sim DA, Keane PA, Mehta H, Fung S, Zarranz-Ventura J, et al (2013) Repeatability and Reproducibility of Choroidal Vessel Layer Measurements in Diabetic Retinopathy Using Enhanced Depth Optical Coherence Tomography. Investigative Ophthalmology & Visual Science 54: 2893–2901. 23538058 10.1167/iovs.12-11085

